# Rapid detection of mpox virus using recombinase aided amplification assay

**DOI:** 10.3389/fcimb.2023.1008783

**Published:** 2023-02-23

**Authors:** Xiaohu Cui, Bing Du, Junxia Feng, Yanling Feng, Jinghua Cui, Chao Yan, Hanqing Zhao, Lin Gan, Zheng Fan, Tongtong Fu, Ziying Xu, Rui Zhang, Shuheng Du, Yao Zhou, Ziyan Tian, Qun Zhang, Hanyu Fu, Guanhua Xue, Jing Yuan

**Affiliations:** ^1^ Department of Bacteriology, Capital Institute of Pediatrics, Beijing, China; ^2^ School of Biological Sciences, The University of Edinburgh, Edinburgh, United Kingdom

**Keywords:** Mpox virus, molecular diagnostic method, recombinase-aided amplification, RAA assay, rapid detection

## Abstract

A recent, unprecedented outbreak of human mpox virus infection has led to cases in non-African nations, and the number of confirmed or suspected cases outside of Africa has exceeded 1,000 within 5 weeks. Mpox may pose a double threat to public health in the context of the ongoing COVID-19 pandemic. It is difficult to distinguish mpox virus infection from other diseases in the early stages, and patients are contagious from the onset of nonspecific symptoms; therefore, it is crucial to develop rapid and specific diagnostic methods. The diagnosis of mpox relies on real-time polymerase chain reaction, a time-consuming method that requires a highly sophisticated thermal cycler, which makes it unsuitable for widespread use in underdeveloped areas, where the outbreak is still severe. In this study, we developed a recombinase-aided amplification (RAA) assay that can detect mpox virus within 5–10 minutes. The conserved regions of the A27L gene and F3L gene were selected as targets, as they amplify well from different mpox virus clades with no cross-reaction from other pathogens. The sensitivity of this RAA assay is 10 copies/reaction for the A27L gene and 10^2^ copies/reaction for the F3L gene. When applied to simulated clinical samples, both targets showed 100% specificity, and the detection limits were consistent with the sensitivity results. Moreover, through clinical blinded sample detection, RAA exhibits the same detection power as RT-PCR. In summary, the RAA mpox assay described here exhibits rapid detection, high sensitivity and specificity, and low operational difficulty, making it suitable for mpox virus detection in less developed countries and regions.

## Introduction

Mpox, a zoonotic disease caused by an orthopoxvirus, is generally considered to be endemic only to Africa (Bunge et al., 2022). However, a recent, unprecedented outbreak of human mpox virus has resulted in the detection of cases in Europe, the Americas, and Australia (Walter and Malani, 2022). Since the first mpox case was reported by the UK Health Security Agency on May 7, the number of confirmed or suspected cases to emerge in non-African nations has exceeded 1,000 within 5 weeks (Kozlov, 2022; Kraemer et al., 2022). Epidemiological investigations to date have shown that these cases have no substantial association with travel to endemic areas (World Health Organization, 2022), while preliminary genomic sequence data are nearly identical (Kozlov, 2022), suggesting that these cases are due to rapid person-to-person transmission of the mpox virus outside of Africa. It is still unclear whether this striking increase in cases is because of changes in the transmission characteristics or virulence of mpox viruses, but it is worth noting that the mpox epidemic may pose a double threat to public health in the context of the ongoing COVID-19 pandemic.

The incubation period for mpox ranges between 5 and 21 days (Kozlov, 2022). Clinical symptoms consist mainly of a rash with distinct skin lesions, accompanied by fever, myalgia, and lymphadenopathy (Guarneri et al., 2022). Patients are likely to be contagious from the onset of symptoms until all lesions have resolved, and during this period mpox virus transmission can occur through direct and close contact *via* droplets, bodily fluids, or fomites (Guarneri et al., 2022). However, it is difficult to make a differential diagnosis with other infectious diseases such as chancroid, varicella-zoster, herpes simplex, and hand-foot-and-mouth disease; therefore, it is important to develop rapid and specific diagnostic methods to identify and control the spread of the disease at the early stage.

Presently, the diagnosis of mpox relies on real-time polymerase chain reaction (RT- PCR) analysis of skin exudate samples, nasopharyngeal swabs, or sputum samples (Kozlov, 2022). However, RT-PCR is time-consuming and requires highly specialized equipment, which makes it difficult to carry out on a large scale in less developed countries. Recombinase-aided amplification (RAA), a rapid and efficient isothermal amplification technique, can detect specific target genes within 10-30 min at 39°C (Xue et al., 2020a). This technology has been used in the clinical detection of pathogens such as SARS-CoV-2, noroviruses, hepatitis B virus, and other pathogens (Xue et al., 2020a; Xue et al., 2020b; Qin et al., 2021; Fan et al., 2022).

In this study, we developed an RAA assay that detects mpox virus within 5–10 min with extremely high sensitivity and specificity. Rapid detection, low cost, and low operational difficulty may make this assay suitable for mpox virus detection in less developed countries and regions.

## Methods

### Primer and probe design

The mpox virus sequences were downloaded from the National Center for Biotechnology Information (NCBI) GenBank database (https://www.ncbi.nlm.nih.gov/). The conserved regions were used to manually design the primers and probes. The specificity of the primers and probes was confirmed using the Primer-BLAST function of NCBI. The online software program OligoEvaluator (http://www.oligoevaluator.com) was used to check for and avoid sequences leading to the formation of primer dimers or hairpin structures. All primers and probes were synthesized and purified by Sangon Biotech (Shanghai, China) using high-performance liquid chromatography.

### Pseudovirus and plasmid construction

Pseudovirus constructed from replication-deficient human type 5 adenovirus (Ad5) and the mpox virus A27L/F3L gene (GenBank accession no. ON563414) was purchased from Sangon Biotech (Shanghai, China). Genomic regions from variola virus (GenBank: LR800245.1: 132045 -132564;42768 -43236), cowpox virus (GenBank: MK035759.1:152951- 153490; 63463 - 63931), and vaccinia virus (GenBank: MT227314.1:140022 -140515; 50618-51086) were synthesized and cloned into the vector pUC57. The pseudovirus was diluted 10-fold to concentrations ranging from 10^7^ copies/μL to 10^0^ copies/μL and stored at − 80 °C until use.

### DNA isolation

DNA was extracted from pseudovirus, bacteria, and clinical samples using a QIAamp DNA Mini Kit (Qiagen, Hilden, Germany) and then stored at − 80 °C for use.

### Recombinase-aided amplification assay

The RAA assays were performed following the protocol provided with a commercial RAA kit (Jiangsu Qitian Bio-Tech Co., Ltd., China). The reaction mixtures contained reaction buffer (25 µL), DNase-free water (15.7 µL), 10 µM primer F, (2.1 µL), 10 µM primer R (2.1 µL), DNA template (2 µL), 280 mM magnesium acetate (2.5 µL), 10 µM probe (0.6 µL), and extracted DNA template (2 µL). The tubes were placed in a B6100 Oscillation mixer (QT-RAA-B6100, Jiangsu Qitian Bio-Tech Co. Ltd., China) and incubated for 4 min, then mixed briefly and centrifuged, and finally transferred to a fluorescence detector (QT-RAA-1620, Jiangsu Qitian Bio-Tech Co. Ltd., China) and measured for 20 min at 39 °C.

### Sensitivity and specificity of the RAA assay

The analytical sensitivity of the RAA assay was determined using 10-fold serial dilutions of the pseudovirus ranging from 10^5^ to 10^0^ copies/µL, as calculated using the following formula: DNA copy number (copy number/µL) = [6.02 × 10^23^ × nucleic acid concentration(ng/µL) × 10^-9^]/[DNA length (in nucleotides) × 660], and then stored at −80°C until use. The assay specificity was evaluated by testing other common pathogens, including coxsackievirus A16, enterovirus A71, respiratory syncytial virus (RSV, type A and B), influenza B viruses, human metapneumovirus, human coronaviruses, human bocavirus, human rhinovirus, *Mycoplasma pneumonia*, *Klebsiella pneumoniae*, *Haemophilus influenzae*, and *Staphylococcus aureus.* The above pathogens are clinically isolated and stored in our laboratory. Nucleic acids were extracted using a QIAamp DNA Mini Kit (Qiagen, Hilden, Germany). At least 20ng/μL of pathogenic nucleic acid was added to each reaction system. To test the specificity with regards to other orthopoxviruses, the plasmid containing homologous regions of variola virus, cowpox virus, and vaccinia virus was used. 10^5^ copies/µL of plasmid were added to each reaction system as calculated using the formula mentioned above.

### Detection efficacy of the RAA assay in different kinds of simulated clinical samples

A total of 84 clinical samples (24 skin exudate samples, 18 nasopharyngeal swabs, 18 sputum samples, 12 urine samples and 12 rectal swab samples) were collected from patients and healthy people. In order to determine the influence of different sample types on the detection efficacy, 10^7^ to 10^0^ copies/µl of the pseudovirus were added to the sample prior to DNA extraction. Each detection was performed in triplicate.

### Real-time PCR assay

RT-PCR targeting F3L and A27L was performed for comparison with the RAA detection method. The reaction system contained 2x qPCR Mix and 10 µM each of forward primer, reverse primer, probe, and template DNA. The cycling conditions were as follows: heating at 50°C for 20 min, then heating at 95°C for 5 min, followed by 40 cycles of denaturation at 95°C for 5 s and annealing/extension at 60°C for 15 s. A threshold cycle (Ct value) < 38 was considered to indicate a positive sample.

### Detection efficacy of the RAA assay in clinical samples

We obtained 10 clinical samples from leision swab of pus, which were provided by Randox company and evaluated the efficacy of this method by performing both RAA and RT-PCR assays. The kappa and p-values of the RAA and RT-PCR assays (with sequencing) were calculated. The statistical analysis was conducted with SPSS 21.0(IBM, Armonk, NY, United States). The present study was performed in compliance with the Helsinki Declaration (Ethical Principles for Medical Research Involving Human Subjects) and was approved by the research board of the Ethics Committee of the Capital Institute of Pediatrics. Each detection was performed in triplicate.

## Results

### RAA assay primer and probe design

**Figure 1:**
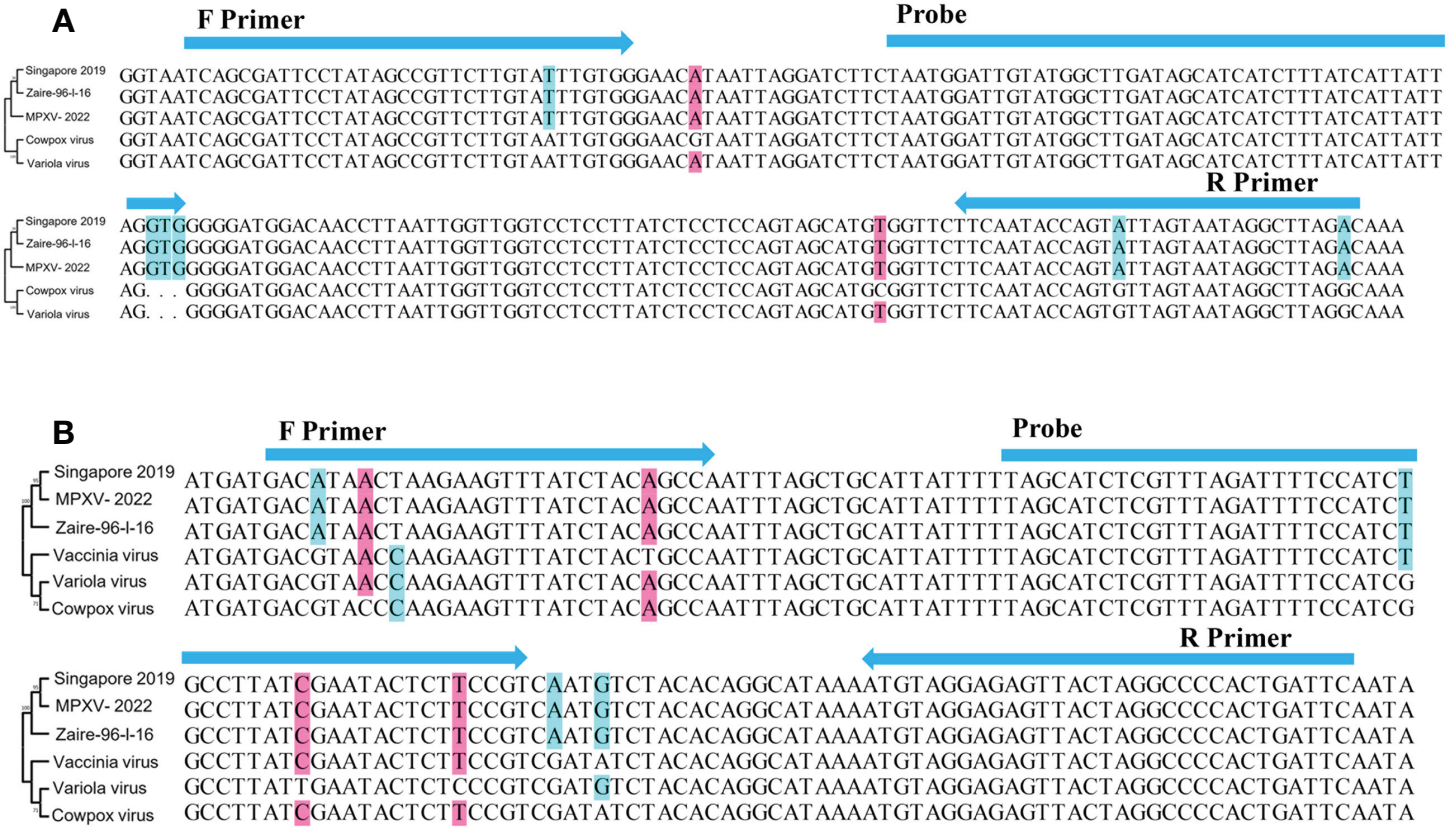
Schematic diagram of the relative position of primers and probes. **(A)** A27L gene and **(B)** F3L gene. Singapore 2019(GenBank: MT250197.1), Zaire-96-I-16(GenBank: AF380138.1), Variola virus(GenBank: LR800245.1), Cowpox virus(GenBank: MK035759.1); Vaccinia virus(GenBank: MK314713.1). Phylogenetic trees were constructed by MEGA 7 (Kumar et al., 2016), using the maximum likelihood method,based on A27L gene and F3L gene, respectively.

A27L and F3L were selected as target genes. Given the homology between mpox viruses and other viruses, we selected gene sequences corresponding to a Congo Basin branch strain (mpox virus strain Zaire-96-I-16, GenBank: AF380138.1), a West African strain (mpox virus strain Singapore 2019, GenBank: MT250197.1), variola virus (GenBank: LR800245.1), cowpox virus (GenBank: MK035759.1), and vaccinia virus (GenBank: MK314713.1), compared the sequences, and constructed phylogenetic trees. The mpox viruses segregated to one branch, which shows that the two genes have high homology among mpox viruses and can be used to distinguish mpox virus from other orthopoxviruses. The primers and probes were manually designed based on the conserved regions of these two genes (**Figure 1** and **Table 1**).

**Table 1:** Sequences of the primers and probes used for the RAA assay.

Gene	Primer/Probe	Sequence (5′–3′)	
A27L for RAA	Forward	TCAGCGATTCCTATAGCCGTTCTTGTATTTGTG	This study
	Probe	CTAATGGATTGTATGGCTTGATAGCATCATC[FAM-dT] [THF][BHQ-dT] ATCATTATTAGGTGG	
	Reverse	GTCTAAGCCTATTACTAATACTGGTATTGA	
A27L for Realtime PCR	Forward	GCGACTTCAGGAGTTAGTAGAAG	This study
	Probe	[FAM]CAACGCTGGAATCGATACTCAAGTTAAGGA[BHQ]	
	Reverse	CGGATGATGACGATGAGGTATT	
F3L for RAA	Forward	GACATAACTAAGAAGTTTATCTACAGCCAATTTAGC	This study
	Probe	TAGCATCTCGTTTAGATTTTCCATCTGCCT[FAM-dT] [THF][BHQ-dT] CGAATACTCTTCCGT	
	Reverse	GAATCAGTGGGGCCTAGTAACTCTCCTACA	
F3L for Realtime PCR	Forward	CTCATTGATTTTTCGCGGGATA	Reference (Kulesh et al., 2004)
	Probe	[FAM]CATCAGAATCTGTAGGCCGT[MGB]	
	Reverse	GACGATACTCCTCCTCGTTGGT	

FAM, 6-carboxyfluorescein; THF, tetrahydrofuran; BHQ, black hole quencher; MGB, Minor Groove Binder.

### Analytical specificity of the RAA assay

**Figure 2:**
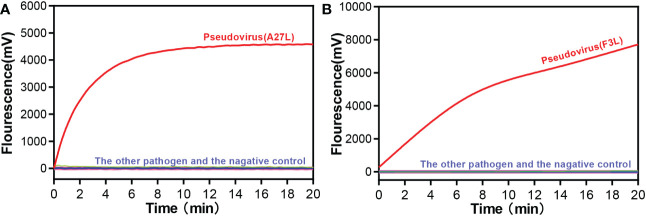
Specificity of the RAA assay for **(A)** A27L gene and **(B)** F3L gene of Mpox virus detection. Only the pseudovirus produced amplification signals, whereas the other pathogen samples and the negative control produced no amplification signals.

We selected common respiratory and intestinal pathogens, such as coxsackievirus A16 and enterovirus A71, which can cause the similar symptoms to mpox virus, to test the ability of the RAA assay to assist in clinical diagnosis. Other orthopoxvirus viruses were also tested for specificity. As shown in **Figure 2**, only the mpox virus produced amplification signals, while the other samples tested negative. It meaned, the RAA assay demonstrated high specificity for the detection of mpox virus (100%).

### Analytical sensitivity of the RAA assay

**Figure 3:**
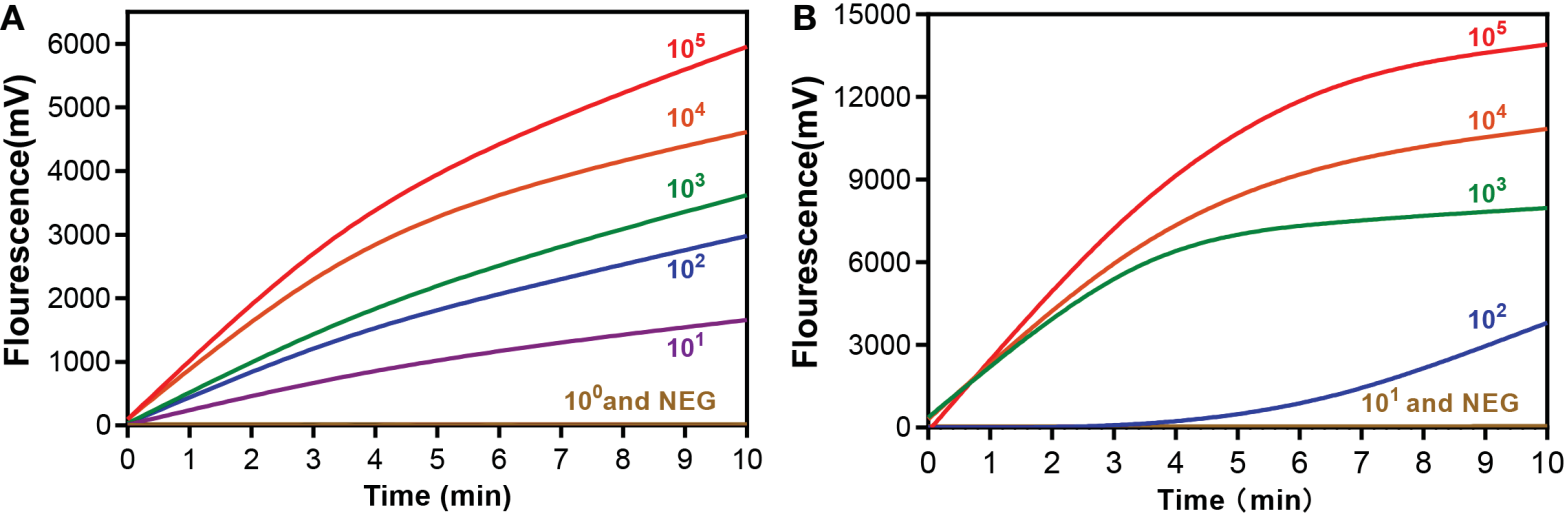
Sensitivity of the RAA assay for **(A)** A27L and **(B)** F3L gene of mpox virus detection. A serial dilution of the pseudovirus was used ranging from 10^5^to 10^0^ copies/reaction. A negative control (replace the pseudovirus with ddH_2_O) was also assayed.

The sensitivity of the RAA assay for mpox detection was determined using a panel of serially diluted solutions of pseudovirus containing the A27L gene or the F3L gene. As shown in **Figure 3**, the RAA assay can detect samples with a minimum of 10 copies/reaction for the A27L gene, while the lowest detectable value was 10^2^ copies/reaction for the F3L gene.

### Efficacy of the RAA assay detecting mpox virus in simulated clinical samples

To test the detection efficacy of the established method in clinical specimens, a total of 84 different clinical samples (skin exudate samples, nasopharyngeal swabs, sputum samples, urine samples and rectal swab samples) were collected from 12 patients, all of whom had *Staphylococcus aureus* infection, 10-20 years of age and had 5-20 skin lesions and 24 healthy volunteers. Pseudovirus was added to these samples prior to DNA extraction. The detection limit of the RAA assay in these simulated clinical samples was 10 copies/reaction for the A27L gene and 10^2^ copies/reaction for the F3L gene. The lower limit of detection was consistent across samples. None of the negative samples yielded a false-positive result. All these detection results were consistent with the RT-PCR results (**Table 2**). And the clinical specificity of the RAA assay was calculated as 100%.

**Table 2:** Lower limits of detection in different kinds of simulated clinical samples.

simulated clinical sample type	F3L (copies/reaction)		A27L (copies/reaction)	
	RAA	RT- PCR	RAA	RT- PCR
skin exudate	10^2^	10^2^	10^1^	10^1^
nasopharyngeal swabs	10^2^	10^2^	10^1^	10^1^
sputum	10^2^	10^2^	10^1^	10^1^
urine	10^2^	10^2^	10^1^	10^1^
rectal swab	10^2^	10^2^	10^1^	10^1^

### Detection efficacy of the RAA assay in clinical samples

**Figure 4:**
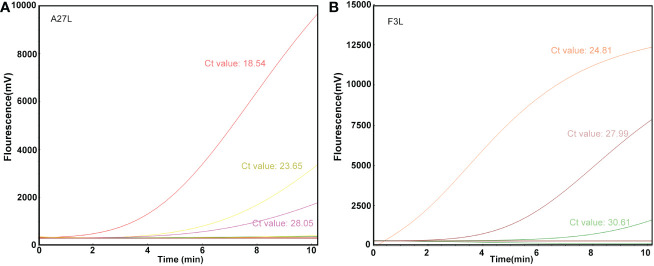
Detection efficacy of the RAA assay in clinical samples. The clinical blinded samples were detected using RAA and RT-PCR assays at the same time, and the results of A27L **(A)** and F3L **(B)** were shown respectively, and the values next to the curves were results of RT-PCR assay.

The RAA assay was then evaluated with 10 blind samples, and the results were verified by RT-PCR. From these 10 blind samples, 3 were positive for mpox (**Figure 4**). All the results were 100% consistent with the results from the RT-PCR assay and the Kappa value is calculated as 1. No significant differences between the detection results from RAA and RT-PCR were observed.

## Discussion

The outbreak of mpox virus infections in non-African nations may pose a double threat to public health in the context of the ongoing COVID-19 pandemic. Mpox virus infection is difficult to distinguish from other diseases such as hand-foot-and-mouth disease and herpes in the early stages, and patients are contagious from the onset of nonspecific symptoms (Walter and Malani, 2022). Diagnosis is based primarily on clinical examination; therefore, a rapid and sensitive auxiliary detection technology is urgently needed.

RT-PCR, is a time-consuming method that requires a highly sophisticated thermal cycler (Kulesh et al., 2004). Although outbreaks of mpox in non-African countries have aroused public alarm, there are also many cases of mpox in African regions, where sanitary and economic conditions mean that highly specialized RT-PCR equipment is not in widespread use. The traditional RT-PCR technology costs about $45/sample and the detection cycle is more than 2 hours (Pliakos et al., 2018), which means that RT-PCR is not suitable for large-scale screening and real-time monitoring. RAA is an isothermal amplification technique that does not require a classic thermostable enzyme or a sophisticated thermal cycler (Xue et al., 2020a), it has a lower cost ($5/sample), faster detection speed (~10 min) and simpler reaction conditions, not requiring complex instruments, making it more suitable for use in less developed countries and regions.

Mpox virus has two known distinct clades, one of which is endemic to west Africa and one of which is primarily found in the Congo Basin. The Congo Basin branch strains, or known as Clade one (I), can cause more severe disease (Li et al., 2010), resulting in a mortality rate of about 10%, while the mortality rate of the West African branch strains, or known as Clade two (II), is about 1% (Chen et al., 2005; Sklenovska and Van Ranst, 2018). Sequence comparison has shown that the currently circulating virus belongs to the Clade two (II) (Saijo et al., 2009). Some researchers have selected E9L, B6R, G2R, C3L, and other genes as targets for detecting mpox virus (Davi et al., 2019; Guarneri et al., 2022). Here, we chose A27L and F3L as target genes, as the phylogenetic tree (**Figure 1**) showed that these two genes clearly distinguished mpox viruses from other viruses. Two sets of primers and probes were designed based on the conserved regions of these two genes. These primers and probes showed extremely high specificity (100%) for mpox virus and amplified both Clade one (I) and Clade two (II) strains, with no cross-reaction with other pathogens. The analytical sensitivity of this assay was 10 copies/reaction for the A27L gene and 10^2^ copies/reaction for the F3L gene in simulated clinical samples, whereas the sensitivity of a previously reported real-time PCR assay was 3.5–100 copies (Kulesh et al., 2004; Li et al., 2006; Li et al., 2010), suggesting that the RAA assay has a similar detection sensitivity.

As mpox virus is thought to spread by close contact with bodily fluids (Walter and Malani, 2022),we added two types of pseudovirus at different concentrations to skin exudate samples, nasopharyngeal swabs, sputum samples, urine samples and rectal swab samples to generate simulated clinical samples that were then evaluated using the RAA assay. By estimating the theoretical sample copy number, we found that the minimum detected copy numbers in these types of clinical samples was consistent with the sensitivity results. And all these detection results were consistent with the RT-PCR results (**Table 2**). This means that the sample type has little effect on the detection efficiency of the RAA. In addition, none of the clinical samples that did not contain pseudovirus tested positive, demonstrating the high specificity of the RAA assay (100%).

Since the outbreak of mpox last year, many detection technologies have been rapidly developed. Chelsky et al. developed a real-time PCR protocol for direct detection without DNA extraction, the reliable minimum detection concentration is 50 copies/mL, the cycle time is 35 minutes, and the cost is decreased to about $16 (Chelsky et al., 2022). Another research team’s modified high-throughput PCR testing sensitivity was 4.795 (95% CI 3.6-8.6) copies/mL (Norz et al., 2022). Although these two studies have improved sensitivity and reduced some costs, they still rely on expensive instruments and are not suitable for rapid screening or application in undeveloped areas. In addition, studies have also been done to detect the mpox virus using isothermal amplification technique. Mao et al. developed RAA technology that targets the G2R gene, showing high sensitivity (10^0^ copies/reaction), and the results can be visualized within 20-30 min (Mao et al., 2022). Although the sensitivity of the RAA protocol targeting A27L and F3L did not reach 10^0^ copies/reaction, it had an earlier peak time, which suggests that using A27L and F3L as dual targets may show higher assay performance. Other types of thermostatic amplification techniques (such as loop-mediated isothermal amplification method) show similar sensitivity as our studies (Feng et al., 2022).

In addition, there are some shortcomings in this study, limited by the availability of clinical samples, the experiment only used only 10 blind samples to evaluate the detection efficacy. And only two target genes of mpox virus were detected, no internal control was added for quality supervision. Also, two- tube assay for two genes were not performed. The efficacy of this method also needs to be further clinically tested.

In summary, the mpox RAA assay developed here has high specificity and sensitivity and provides a simple and reliable method for mpox virus detection. Rapid detection, low cost, and low operational difficulty may make it suitable for use in less developed countries and regions.

## Data availability statement

The original contributions presented in the study are included in the article/Supplementary Material. Further inquiries can be directed to the corresponding authors.

## Author contributions

JY and GX designed the experiments and revised the manuscript. XC, BD, GX, JF, QZ, RZ, and YZ performed the experiments. JC, CY, HZ, and YF collected the clinical samples. SD, LG, ZF, TF, ZX, and YZ analyzed the results. XC and GX wrote the manuscript. All authors contributed to the article and approved the submitted version.
